# Aldehyde dehydrogenase and estrogen receptor define a hierarchy of cellular differentiation in the normal human mammary epithelium

**DOI:** 10.1186/bcr3663

**Published:** 2014-05-27

**Authors:** Gabriella Honeth, Sara Lombardi, Christophe Ginestier, Minhee Hur, Rebecca Marlow, Bharath Buchupalli, Ireneusz Shinomiya, Patrycja Gazinska, Silvia Bombelli, Vernie Ramalingam, Anand D Purushotham, Sarah E Pinder, Gabriela Dontu

**Affiliations:** 1 Stem Cell Group, Research Oncology, King’s College London School of Medicine, London SE1 9RT, UK; 2 Centre de Recherche en Cancérologie de Marseille, Inserm, CRCM, U1068, Laboratoire d’Oncologie Moléculaire, Marseille F-13009, France; 3 Department of Internal Medicine, University of Michigan, Ann Arbor MI 48109, USA; 4 Department of Surgery, Cheil General Hospital & Women’s Healthcare Center, Kwandong University College of Medicine, Seoul 100-380, South Korea; 5 Breakthrough Breast Cancer Research Unit, King’s College London School of Medicine, London SE1 9RT, UK; 6 Department of Health Sciences, University of Milano-Bicocca, 20900 Monza, MB, Italy; 7 Research Oncology, King’s College London School of Medicine, London SE1 9RT, UK

## Abstract

**Introduction:**

Although estrogen and progesterone play a key role in normal mammary development and in breast cancer, the potential for proliferation and lineage differentiation as well as origin of cells that express the estrogen receptor (ER) in normal breast epithelium are not known. Some evidence suggests that normal human mammary stem/progenitor cells are ER–, but the identity of these cells and the cellular hierarchy of breast epithelium are still subjects of controversy. It is likely that elucidation of these aspects will bring insight into the cellular origin of breast cancer subtypes.

**Methods:**

We used fluorescence-activated cell sorting of primary human mammary epithelial cells along with *in vitro* and *in vivo* functional assays to examine the hierarchic relation between cells with aldehyde dehydrogenase enzymatic activity (ALDH+ cells) and ER+ cells in the normal human breast epithelium. We assessed the proliferation and lineage differentiation potential of these cells *in vitro* and *in vivo*. A gene reporter assay was used to separate live ER+ and ER– mammary epithelial cells. With shRNA-mediated knockdown, we investigated the role of ALDH isoforms in the functionality of mammary epithelial progenitor cells.

**Results:**

We describe a cellular hierarchy in the normal human mammary gland in which ER–/ALDH+ cells with functional properties of stem/progenitor cells generate ER+ progenitor cells, which in turn give rise to cells of luminal lineage. We show that the ALDH1A1 isoform, through its function in the retinoic acid metabolism, affects the proliferation and/or early differentiation of stem/progenitor cells and is important for branching morphogenesis.

**Conclusions:**

This study presents direct evidence that ER+ cells are generated by ER–/ALDH+ stem/progenitor cells. We also show that ER+ cells are able to generate cell progeny of luminal lineage *in vitro* and *in vivo*. Loss of ALDH1A1 function impairs this process, as well as branching morphogenesis and clonogenicity in suspension culture. This latter effect is reversed by treatment with retinoic acid.

## Introduction

Unlike other tissues, the mammary gland tissue undergoes morphogenesis postnatally, a process most likely sustained by mammary stem and progenitor cells and controlled by systemic factors such as the ovarian steroid hormones estrogen and progesterone. Studies in transgenic mice have demonstrated that estrogen receptor alpha (ER) and progesterone receptor (PR) are critical for mammary morphogenesis [1,2]. PR is a transcriptional target of ER, and these two receptors show tight co-expression in normal mammary epithelial cells [3]. Although mammary stem cells appear not to express the steroid receptors ER and PR, their relative number increases in response to progesterone, through paracrine signaling involving amphiregulin, Wnt, and Receptor Activator of Nuclear factor Kappa-B Ligand (RANKL) [4-10].

Sustained exposure to steroid hormone levels is a well-established risk factor for breast cancer, supported by numerous epidemiologic studies [11]. Although the majority of breast cancers are ER+, and hormonal intervention is largely used to prevent disease recurrence and/or progression, the mechanisms through which estrogen and progesterone contribute to malignant transformation of mammary epithelium are poorly understood. It is believed that elucidating the cellular hierarchy of differentiation in the normal breast epithelium and understanding the mechanisms by which ovarian steroid hormones control the proliferation of mammary stem/progenitor cells would give insights into the origin of different subtypes of breast cancer.

In the present study, we investigated the hierarchic relation between stem/progenitor cells and ER+ cells, as well as the ability of this latter cell population to proliferate *in vitro* and *in vivo*. We used aldehyde dehydrogenase (ALDH) activity as a marker of stem/progenitor cells in the normal human mammary epithelium. In a previous study, we demonstrated that normal breast cells with high ALDH activity have the broadest lineage-differentiation potential and the highest ability to generate outgrowths *in vivo*, when compared with the rest of the mammary epithelial population [12]. We now show that these cells are ER–, but they give rise to ER+ cells, which in turn can generate cells committed to the luminal lineage. Mammosphere-initiating cells have the highest ability to generate human mammary outgrowths *in vivo*, in xenotransplantation models [13], and are widely used in functional assays specific for stem/progenitor cells. We show that mammosphere-initiating cells are ER–, but generate *in vitro* ER+ and PR+ cells.

We further show that the ALDH isoform ALDH1A1 has a functional role in proliferation of mammary progenitor cells and in branching morphogenesis, through its role in retinoic acid metabolism. Based on these findings and previous knowledge about the function of aldehyde dehydrogenases and retinoic acid in developmental processes, we propose a model for the role of ALDH1A1+ and ER+ mammary epithelial cells in proliferation of adult human mammary epithelium.

## Methods

### Dissociation of normal breast epithelium

Normal breast tissue was obtained with informed consent from patients undergoing mammoplasty for aesthetic or prophylactic reasons, under protocols approved by the IRB and by Guy’s Research Ethics Committee, in compliance with the Human Tissue Act. The tissue was processed as previously described [12]. To generate a single-cell suspension for the *in vivo* studies, a shorter 6-hour collagenase digestion was used. Pieces of tissue were fixed in formalin for 24 to 48 hours before being processed and embedded in paraffin.

### ALDEFLUOR assay and flow sorting

The ALDEFLUOR kit (StemCell Technologies Vancouver, Canada) was used according to manufacturer’s protocol. Cells were sorted by using a FACS Aria II (BD Biosciences, San Jose, CA) with 130-μm nozzle. Cell viability was assessed with LIVE/DEAD Fixable Violet Dead Cell Stain (Life Technologies, Carlsbad, CA). Sorted cells were cytospun onto glass slides for immunofluorescent analysis.

### Immunostaining of cytospins

Cells were fixed with methanol for 20 minutes, washed with PBS, treated with 0.1% Triton X-100 for 5 minutes, and incubated in blocking buffer (PBS with 2% BSA) for 1 hour and subsequently stained with primary antibodies against ALDH1A1 (BD Biosciences, clone 44/ALDH, 1:50) and ALDH1A3 (Santa Cruz Biotechnology, Dallas, TX, clone C-13, 1:200) followed by secondary antibodies anti-mouse AlexaFluor-488 and anti-goat AlexaFluor-555 (Life Technologies, 1:500). Antibody incubations were done for 1 hour in blocking buffer. Nuclei were counterstained with DAPI.

### Immunohistochemistry of paraffin sections

Paraffin-embedded sections (3 μm) of primary or xenotransplanted normal breast epithelium were deparaffinized in xylene and rehydrated in graded alcohol. Antigen retrieval was achieved by heating slides in citrate buffer (Dako Glostrup, Denmark) according to recommendations. Sections were blocked with 10% donkey serum for 1 hour before incubation with primary antibodies in 10% donkey serum for 1 hour at RT. Antibodies used were ALDH1A1 and ALDH1A3 as above, ER raised in either rabbit (Novocastra (Leica) Wetzlar, Germany, 1:100) or in mouse (Dako, 1:100), SMA (Novocastra, 1:100), CK18 (Novocastra, 1:20), Ki67 (Dako, 1:100), MCM2 (Novocastra, 1:50), and RARα (Abcam Cambridge, UK, 1:250). Primary antibodies were detected either with fluorescent secondary antibodies (from Life Technologies, conjugated with AlexaFluor-488 or -555) or enzymatically by using Peroxidase Histostain-Plus Kit (Zymed South San Francisco, CA) or EnVision G2 Doublestain System (Dako), according to the manufacturer’s protocols. Nuclei were counterstained with DAPI and hematoxylin, respectively. For assessing the percentage of ALDH1A1+ and ALDH1A3+ cells detected by IHC, double immunostained tissue sections of normal breast (four different patients) were scanned by using the Hamamatsu Nanozoomer and analyzed by using Digital Images HUB (SlidePath system, Leica). Low-magnification images were used to delineate areas of epithelium. ALDH1A1+ cells, ALDH1A3+ cells, and total number of cells in each nonoverlapping area were counted. Each tissue section contained between 22,000 and 36,500 cells.

### Immunostaining for ER and flow cytometry

ALDEFLUOR-sorted cells were fixed in methanol and stained with antibody against ER (Thermo Scientific, Waltham, MA,, 1:100) followed by FITC conjugated anti-rabbit secondary (Jackson Laboratory West Grove, PA, 1:250). Antibody stainings were done for 20 minutes on ice in Hanks Balanced Salt Solution (HBSS, Gibco Life Technologies) supplemented with 2% FBS. Staining for viability was done by using 1 μg/ml propidium iodide (PI, Sigma St Louis, MO) for 5 minutes.

### ER reporter assay

The ER reporter system, a generous gift from Dr. Polyak, Dana Farber Cancer Institute, was used to sort ER+ cells from normal mammary epithelium and was tested for specificity and sensitivity on ER+ and ER– breast cancer cell lines (MCF7 and MDA-MB-231). It consists of an adenovirus encoding GFP, under the control of 25 estrogen-responsive elements (EREs) and the rat progesterone promoter [14]. Cells were grown in phenol red-free, charcoal-stripped serum containing medium for 8 hours, after which the adenovirus was added in serum-free phenol red-free medium for an additional 15 hours. The medium was then removed and replaced with medium containing serum and β-estradiol (10^−9^*M*) for 48 hours. GFP expression was monitored microscopically. Flow-cytometry analysis and sorting was performed. Previous *in vivo* transplantation studies from the Werb [15] and Visvader [16] groups showed that this short *in vitro* cultivation and viral cell marking do not affect the potential and cell fate of human and mouse mammary epithelial cells [15,16]. An aliquot of the sorted cells was analyzed microscopically for GFP expression and immunostained for ER to test the quality of the sorting. Cells were permeabilized with ice-cold methanol, and stained in suspension with ER and secondary antibodies, as described earlier.

### *In vivo* assays in NOD/scid mice

Xenotransplantation of normal mammary epithelial cells sorted for ALDEFLUOR activity or with the ER reporter in humanized cleared mammary fat pads was performed as previously described [12,17]. For assessing proliferation *in vivo* of ER+ cells, sorted cells were mixed with human mammary fibroblasts in equal ratios, suspended in gelatinous protein (Matrigel, BD Biosciences), and implanted in the cleared and humanized 4th inguinal mammary fat pads (25,000 to 800,000 cells/fat pad). Estrogen pellets (90-day release, 0.5 mg/pellet; Innovative Research of America Sarasota, FL) were implanted subcutaneously at the time of the clearing. Animals were killed after 2 months, and fat pads were fixed in formalin and embedded in paraffin for histologic analysis. The animal studies were performed under protocols approved by UCUCA and the Institutional Committees on Animal Welfare of the United Kingdom Home Office.

### Differentiating culture

Primary human mammary epithelial cells sorted by using the ER reporter were placed on collagen-coated plates at a density of 2,000 viable cells/10-cm-diameter dish. Cells were grown in phenol red-free DMEM-F12 supplemented with 5% charcoal-stripped serum, 5 μg/ml insulin, 1 μg/ml hydrocortisone, 10 ng/ml EGF, 10 ng/ml cholera toxin, and 20 μg/ml gentamycin, and 1×antibiotic-antimycotic. Cells were fixed and immunostained after 12 days.

### Mammosphere culture

Mammosphere culture was performed as previously described [18]. In brief, primary human mammary epithelial cells were plated in ultra-low attachment plates (Corning, Corning, NY) or plates coated with 1% agarose, at a density of 20,000 viable cells/ml in primary culture and 5,000 cells/ml in subsequent passages. Medium used was mammary epithelial basal medium (MEBM, Lonza, Basel, Switzerland) supplemented with 20 ng/ml EGF, 1 μg/ml hydrocortisone, 50 μg/ml insulin, 100 μ*M* 2-mercaptoethanol, 20 μg/ml gentamycin, and 1× antibiotic-antimycotic. The medium did not contain B27 or phenol red. Counting of mammospheres was done manually after 7 to 10 days in six-well plates under a light microscope in at least three wells for each condition. All primary cultures were incubated at 37°C, 10% CO_2_.

### Paraffin embedding of mammospheres

After 7 to 10 days in culture, mammospheres were gently centrifuged at 200 rpm for 2 minutes at RT. The pellet was then resuspended in formalin and allowed to fix for 1 hour at RT. After fixation, mammospheres were centrifuged, and part of the supernatant was removed. An equal volume of melted 4% agarose (Gibco electrophoresis grade) in distilled water was then added to the formalin-fixed pellet (to a final agarose concentration of 2%). The volume was aspirated in a 1-ml syringe on which the top had been cut off and allowed to cool for 30 minutes at RT before the gel was expelled by using the syringe plunger. The gel was wrapped in lens tissue and paraffin/wax processed after routine tissue-processing procedures. Staining of sectioned mammospheres was done as described for paraffin sections.

### ALDH knockdown

Lentiviral vectors (pGIPZ) expressing shRNAs against *ALDH1A1* and *ALDH1A3* (Applied Biosystems, Life Technologies) were used to downregulate the expression of these genes. The vectors were transfected into the 293T packaging cell line by using calcium phosphate precipitate. After 36 hours, supernatants expressing lentivirus were collected, concentrated by using Amicon Ultra Centrifugal Filter Units, 100 kDa (Millipore Billerica, MA), and used to infect normal human mammary epithelial cells in adherent conditions, in serum-free medium. Two days of infection were performed (two cycles each day), at the end of which, cells were checked for GFP expression with fluorescence microscopy. Knockdown (KD) efficiency was determined by using Western blot.

### Western blot

Total cell extracts from KD cells were separated on 10% SDS-PAGE gels and transferred to nitrocellulose membrane (Bio-Rad Hercules, CA). Membranes were blocked with 5% milk and incubated with antibodies against ALDH1A1 (1:1,000) or ALDH1A3 (1:500) in 5% milk in tris-buffered saline (TBS) containing 0.1% Tween overnight at 4°C, followed by incubation with horseradish peroxidase (HRP)-conjugated donkey anti-mouse or -goat secondary antibody (Jackson Laboratories, 1:10,000) for 1 hour at RT. Super Signal West Pico chemiluminescent substrate kit system (Thermo Scientific) was used for signal detection. Membranes were stripped by using 0.2 *M* NaOH, after which they were incubated with antibody against GAPDH (Cell Signaling Technology Danvers, MA, 1:5,000) for loading control. Densitometry of detected bands was done by using ImageJ.

### Matrigel culture

*In vitro* 3D culture of human mammary epithelial cells was performed as previously described [19]. In brief, single-cell suspensions were aggregated overnight in MEBM in 24-well ultra-low-attachment plates at 100,000 cells/well. The next day, TC-treated 24-well plates were coated with 100 μl Matrigel diluted 2:1 in serum-free F12 media (Life Technologies) and allowed to solidify. Approximately 130 aggregates were collected by gentle centrifugation, resuspended in 300 μl Matrigel, diluted 2:1 in F12 containing 5% FBS, insulin (5 μg/ml), hydrocortisone (1 μg/ml), EGF (10 ng/ml), cholera toxin (10 ng/ml), gentamycin (20 μg/ml), and 1× antibiotic-antimycotic, and plated on top of the 100 μl Matrigel in the 24-well plates. Once solidified, 300 μl of F12 with FBS was added on top. Medium was changed every other day.

### Retinoic acid treatment

Mammosphere and Matrigel cultures were treated with 1 μg/ml all-*trans* retinoic acid (Sigma) in DMSO at a final concentration of 0.1%. The same concentration of DMSO was used as control.

### Statistical analysis

Data were analyzed by using GraphPad Prism v6.0. One-way ANOVA followed by the Tukey multiple-comparisons test was performed to determine statistical significance, unless otherwise stated. *P* values <0.05 were considered statistically significant.

## Results

### Normal mammary epithelial cells with high ALDH enzymatic activity are positive for ALDH1A1 or ALDH1A3 by immunostaining

**Figure 1:**
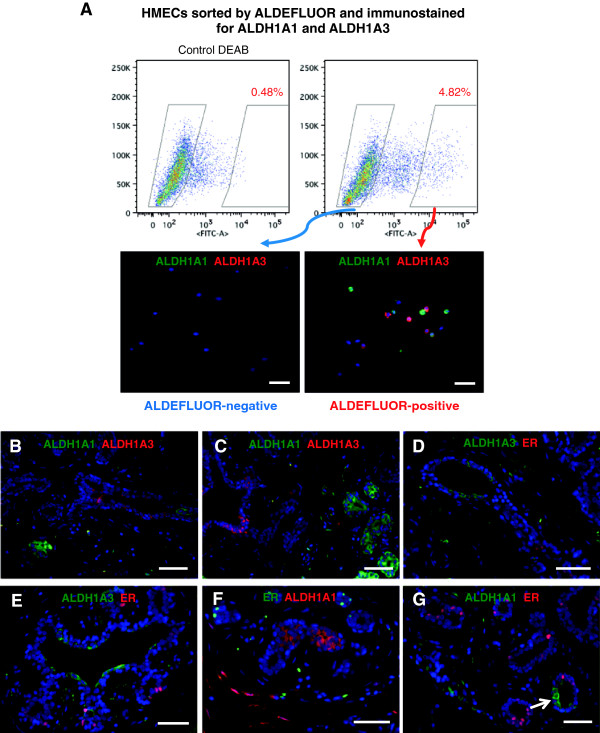
**ALDH+ mammary epithelial cells do not express ER. (A)** Primary human mammary epithelial cells were sorted by ALDEFLUOR (ALDE) positivity, and cytospins with ALDE+ and ALDE– cells, respectively, were stained for ALDH1A1 and ALDH1A3 isoforms. ALDE+ cells were positive for either ALDH1A1 or ALDH1A3 (lower right panel). None of these two isoforms was detectable in the ALDE– fraction (lower left panel). **(B, C)***In situ* staining of normal breast epithelium showed that ALDH1A1 and ALDH1A3 were expressed in distinct cell populations, with no overlap. **(D-G)** No colocalization of ER and ALDH1A3 or ALDH1A1 was observed with immunofluorescence. Nuclear stain (blue DAPI) was performed on all sections. Scale bar = 50 μm.

To identify *in situ* the ALDH+ human mammary epithelial cell population previously shown to be associated with stem/progenitor properties in functional assays, we sought to identify the ALDH isoforms associated with this enzymatic activity. To this end, we used the ALDEFLUOR assay and flow-activated cell sorting (FACS), as previously described [12]. We separated ALDEFLUOR+ and ALDEFLUOR– mammary epithelial cells from three different mammoplasty samples. We subsequently immunostained cytospins from these cell populations for ALDH isoforms. We found that the majority of ALDEFLUOR+ cells expressed either ALDH1A1 (38% ± 17%, mean ± SD) or ALDH1A3 (25% ± 5.7%, mean ± SD) (Figure 1A). No expression of ALDH1A1 and only very-low-intensity levels of ALDH1A3 were detected in a minority of ALDEFLUOR– cells (6% ± 2.5%, mean ± SD). The specificity of antibodies for the ALDH isoforms was tested after knockdown of ALDH1A1 and ALDH1A3, respectively, in cell lines (Additional file 1). The representation of ALDH1A1 and ALDH1A3 cells (cumulated percentages) was similar to the representation of the ALDH+ cell population detected by ALDEFLUOR in three out of four samples (Additional file 2), which further supported that these are the isoforms primarily responsible for the activity, although other isoforms are likely to contribute as well. Consistent with our findings, these two isoforms were found to be significantly elevated in mRNA levels when the ALDEFLUOR+ population was compared with the ALDEFLUOR– fraction in breast cancer cell lines [20]. Both ALDH1A1 and ALDH1A3 have been associated with poor prognosis in breast cancer [12,21-23]. ALDH1A3 was associated with primitive luminal progenitors in normal breast epithelium [24].

IHC analysis of multiple sections of normal breast from six different patients showed that ALDH1A1 and ALDH1A3 were never coexpressed in the same cells in the breast epithelium (Figure 1B, C). High levels of ALDH1A3 were present mostly in larger ducts, and low levels of ALDH1A3 in the smaller extra- and intralobular ductules (Figure 1D, E and Additional file 3D, E). ALDH1A1 was seen in intralobular terminal ductules or at branching points (Figure 1F, G and Additional file 3F-I). Several studies failed to identify an ALDH1A1+ mammary epithelial cell population [24-26]. However, our findings, as well as those from several other groups [27-29], demonstrate unequivocally the existence of this cell population. Considerable differences in technical approach may account for these contradictory results. We henceforth refer to ALDH enzymatically active cells identified by the ALDEFLUOR assay as ALDE+ cells, and to cells identified as positive by immunostaining as ALDH+ (if referring to both isoforms) or ALDH1A1+ and ALDH1A3+, according to the isoform investigated.

### ALDH+ cells in the normal breast epithelium are ER–

Lim *et al*. [25] showed that stem/progenitor cells from the normal human breast identified by flow cytometry as CD49f-high/EpCAM-/Lin- do not express ER. In another study [30], Eirew *et al*. demonstrated that mammary epithelial cells bearing the same markers generated *in vivo* outgrowths that contained ER+ cells. Taken together, these two studies suggest that human mammary stem/progenitor cells are ER– but generate ER+ cells. Similar results were obtained by the Kuperwasser group by using the CD10+/EpCAM– phenotype to identify stem/progenitor cells [31]. We tested whether this conclusion holds true in our experimental approach, which uses ALDH-based markers.

IHC analysis of normal breast epithelium from 11 patients (five to 17 sections examined per sample) showed that neither ALDH1A1 nor ALDH1A3 co-localizes with ER (Figure 1D-G and Additional file 3D-I). In three of the 11 mammoplasty samples analyzed, however, very low levels of ER were detected in a small minority of ALDH1A1+ cells (2.4% ± 0.5% (mean ± SD) ER-low cells in the ALDH1A1+ population, 0.36% ± 0.2 in the total population) (Additional file 3F-J). These may represent a transient, very rare cell population detectable only in a subset of mammoplasty samples. Flow-cytometry analysis of six mammoplasty samples showed no ER expression in ALDE+ cells (Additional file 3A). This was further supported by immunostaining for ER on ALDE-sorted cells (Additional file 3C). These results are consistent with our previous studies in BRCA1 healthy carriers [32].

### ALDE+/ER– cells generate ER+ cells *in vitro* and *in vivo*

**Figure 2:**
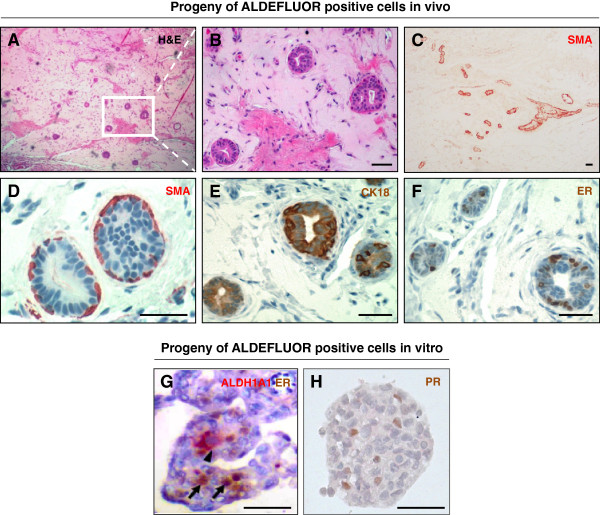
**ALDE+/ER– cells generate ER+ cells *in vitro *and *in vivo*. (A-F)** Ducts generated by the ALDE+ mammary epithelial cells injected in the humanized cleared fat pad of NOD/scid mice: **(A, B)** Fat-pad section stained with H&E. **(C)** Low-magnification image showing section through epithelial duct and emerging branching ductules stained for SMA (AEC, red). **(D-F)** Ductule section stained for myoepithelial marker SMA (**D**, AEC, red), luminal marker CK18 **(E**, DAB, brown), and ER **(F**, DAB, nuclear brown staining). **(G,H)** Mammospheres generated by ALDE+ cells contained ER+ and PR+ cells (DAB, nuclear brown). Double staining for ER and ALDH1A1 showed no overlap between ALDH1A1+ cells (AEC, cytoplasmic red, arrowhead) and the ER+ cells (arrows). Scale bar = 50 μm.

We previously showed that ALDE+, but not ALDE– cells, from the normal breast epithelium generate outgrowths in immunodeficient mice [12]. To test whether ALDE+/ER– cells generate ER+ cells *in vivo*, we sorted ALDE+ cells from four normal mammoplasty samples and implanted 25,000 cells into humanized cleared mammary fat pads of NOD/scid mice. This cell number was chosen to increase the frequency of rare progeny and optimize their detection, based on our previous findings regarding ability of ALDE+ cells to generate outgrowths [12]. As expected, ductal outgrowths were observed in all the transplanted fat pads. Figure 2A and B shows a representative H&E-stained section through a ductal outgrowth generated by ALDE+ cells *in vivo*. Consistent with what we previously described [12], ducts contained two cell layers, an outer layer that stained positive for smooth muscle actin (SMA), a marker of the myoepithelial lineage (Figure 2C, D) and an inner layer that stained positive for the luminal lineage marker cytokeratin (CK) 18 (Figure 2E). ER+ cells were present in outgrowths from all four mammoplasty samples analyzed (Figure 2F). Similar results were obtained *in vitro*. Mammospheres generated by ALDE+/ER– cells contained ALDH1A1+ and ER+ cells (Figure 2G, H).

### ER+ cells proliferate *in vitro* and *in vivo* and generate progeny expressing luminal lineage markers

**Figure 3:**
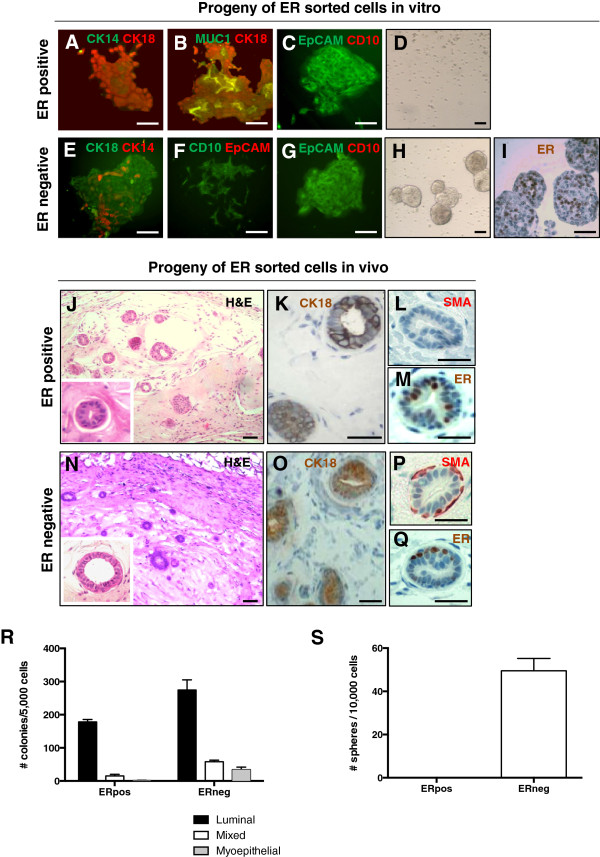
**ER+ cells proliferate *in vitro *and *in vivo *and generate progeny expressing luminal lineage markers. (A-C)** ER+ cells sorted and plated at clonogenic densities on a collagen substratum generated colonies positive for the luminal markers CK18 **(A, B)**, MUC1 **(B)**, and EpCAM **(C)**, and negative for the basal/myoepithelial epithelial markers CK14 **(A)** and CD10 **(C)**. **(D)** ER+ cells did not generate mammospheres. **(E-G)** Sorted ER– cells generated on collagen substrate mixed luminal/basal/myoepithelial colonies composed of CK14+ and CK18+ cells **(E)**, myoepithelial colonies composed of CD10+/EpCAM– cells **(F)**, and luminal colonies composed of CD10–/EpCAM+ cells **(G)**. **(H)** ER– cells generated mammospheres in suspension culture. **(I)** IHC showing ER expression in mammospheres derived from ER– cells. **(J-M)** Representative sections through epithelial ducts generated *in vivo* by ER+ cells. Immunostaining showed positivity for luminal marker CK18 **(K)** but not myoepithelial marker SMA **(L)**. ER+ cells could also be seen **(M)**. **(N-Q)** Representative sections through epithelial ducts generated *in vivo* by ER– cells. Immunostaining showed positivity for both luminal marker CK18 **(O)** and myoepithelial marker SMA **(P)**, as well as for ER **(Q)**. Scale bar = 50 μm. **(R)** Quantitative analysis of types of colonies generated by the ER+ and ER– cell populations. **(S)** Efficiency of primary sphere formation from ER+ and ER– cell populations. ER+ cells did not produce any mammospheres. Representative data from two different mammary samples.

We enquired subsequently whether, in our experimental system, ER+ cells are only “sensory” cells or if these cells also possess progenitor potential, as has been indicated by studies in mice using surrogate markers that enrich for ER+ cells [26,33]. ER+ cells from normal breast epithelium were identified and separated with FACS by using a reporter system built on estrogen responsive elements (ERE) and the rat progesterone promoter (Additional file 4). When plated at clonogenic densities both ER+ and ER– sorted cells generated colonies in adherent conditions (Figure 3A-C, E-G, R), but only ER– cells formed mammospheres in suspension culture (Figure 3D, H-I, S). These mammospheres contained ER+ cells, demonstrating that ER+ cells arise from ER– progenitors. In adherent culture on a collagen substratum, ER+ cells generated progeny bearing only luminal markers (CK18, MUC1, EpCAM) (Figure 3A-C, R) while ER– cells or cells sorted on viability alone generated mixed luminal-myoepithelial colonies containing both CK14+ and CK18+ cells as well as pure luminal (EpCAM+) and myoepithelial (CD10+) colonies (Figure 3E-G, R). The ER– cell population contained bipotent progenitor cells, which generated cell colonies displaying mixed-lineage markers. The ER– cell population and cells sorted on viability alone served as controls, showing that the mammary epithelial cells used in these experiments had the capacity to generate both myoepithelial and luminal cells. The restricted potential of ER+ cells was not due to the culture conditions or the manipulation of cells during sorting.

Additional evidence that ER+ cells can proliferate in culture comes from the colocalization of ER with proliferation marker Ki67 in mammosphere sections (see Additional file 5). In normal breast sections, the percentage of ER+ cells that express proliferation markers is very low [3,34]. In culture, ER expression is downregulated within 4 to 5 days in standard 2D culture [8,35] and within 7 days in Matrigel drip culture [35]. In mammosphere sections, ER expression can be detected after 10 to 12 days in culture (Additional file 5).

To investigate the ability of ER+ cells to proliferate *in vivo*, ER+, ER–, and unseparated live-cell populations were sorted from three different mammoplasty samples and implanted in cleared humanized fat pads of NOD/scid mice (25,000 to 800,000 cells/fat pad). These cell numbers were chosen based on previous findings [12,17] regarding growth potential of mammary epithelial cells in xenografts, taking into account that no enrichment in stem/progenitor cells was expected. All the cell populations generated outgrowths (see Additional file 6), although not in all fat pads transplanted with 25,000 ER+ cells. Outgrowths from ER+ cells had only one layer of CK18+ luminal cells, which contained both ER+ and ER– cells (Figure 3J-M). Myoepithelial markers were not detected in any of the outgrowths from ER+ cells (Figure 3L and data not shown). ER– cells and unseparated cells from the same samples generated outgrowths in all implanted fat pads. These outgrowths were composed of two cell layers, consisting of CK18+ luminal cells surrounded by a layer of SMA+ myoepithelial cells (Figure 3N-P), indicating that the differentiation potential of the cells was not altered by experimental manipulations. These outgrowths contained a subset of ER+ cells (Figure 3Q).

Taken together, these data indicate that the ER– cell population contains a subset of cells that can generate ER+ cells. ER+ cells are able to proliferate and generate outgrowths that are restricted to differentiation along the luminal lineage.

### ALDH enzymatic activity is necessary for mammary stem/progenitor cell proliferation and for branching morphogenesis

**Figure 4:**
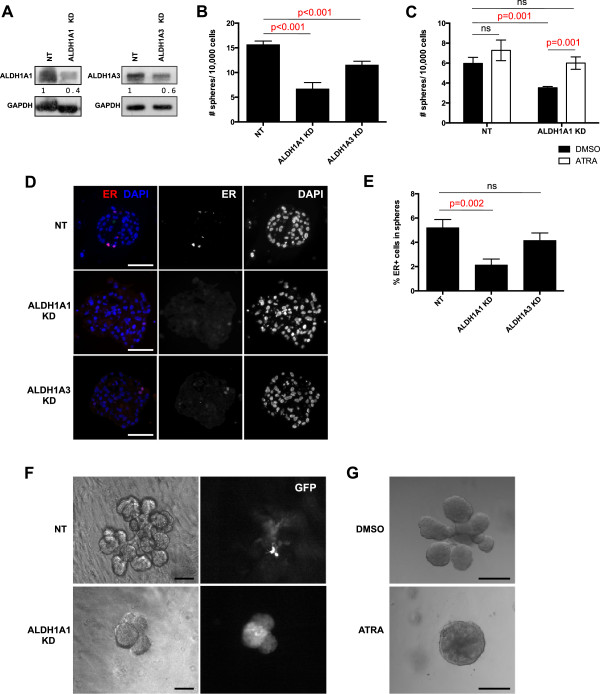
**ALDH activity is important for mammary stem/progenitor cell functions. (A)** Expression of ALDH1A1 and ALDH1A3 in primary human mammary epithelial cells after knockdown (KD) of ALDH1A1 and ALDH1A3, respectively, by using shRNA. **(B)** Effects of ALDH KD on mammosphere formation. Primary human mammary epithelial cells were infected with shRNA against ALDH1A1 and ALDH1A3, respectively, and cells were plated in suspension at low density and allowed to form mammospheres. The numbers of mammospheres formed were counted after 10 days and compared with nontargeting shRNA (NT). Data are presented as mean number of spheres formed/10,000 plated cells ± SD. **(C)** Reduction of mammosphere formation after silencing ALDH1A1 by using shRNA was rescued by treatment with 1 μm all-*trans* retinoic acid (ATRA). **(D)** Expression of ER in mammospheres from cells infected with shRNA against ALDH1A1 and ALDH1A3, respectively. **(E)** Quantification of ER expression in mammospheres where ALDH1A1 or ALDH1A3 has been silenced shows that ER expression was significantly reduced in mammospheres from ALDH1A1 KD cells. **(F)** Effect of ALDH1A1 KD on branching in 3D culture. Cells were infected with shRNA against ALDH1A1 or NT control and embedded in Matrigel. Pictures show representative structures at 13 days of cultivation. **(G)** Effect of retinoic acid treatment on branching. Matrigel cultures were treated with 1 μm ATRA or DMSO control. Pictures show representative structures at 15 days of cultivation. ALDH1A1 KD was obtained by using a pool of two different shRNAs (see Additional file 7) in all these experiments. Scale bar = 50 μm.

To assess whether ALDH activity is necessary for the functionality of mammary stem/progenitor cells, we knocked down ALDH1A1 and ALDH1A3 in primary human mammary epithelial cells by using lentivirus-mediated shRNA interference (Figure 4A). We tested the effect of this downregulation on mammosphere formation. We found that ALDH1A1 knockdown (KD) significantly reduced the number of primary and secondary spheres formed in suspension culture compared with nontargeting (NT) control in five different mammoplasty samples (40% to 90% reduction). ALDH1A3 KD modestly decreased sphere formation (25% reduction) (Figure 4B and Additional file 7).

The biochemical function of ALDH is to convert retinol to retinoic acid (RA); this represents the only source of RA in tissues. Therefore, we tested whether treatment with RA could rescue the KD effect of ALDH1A1. Mammosphere formation in the ALDH1A1 KD cell cultures treated with 1 μm all-*trans* retinoic acid (ATRA) was restored to levels seen in control cultures (Figure 4C). ATRA treatment of control mammary epithelial cells in suspension culture did not affect mammosphere formation.

We also investigated the expression of retinoic acid receptor alpha (RARα) in the mammary epithelium and found that, consistent with previous reports [36,37], RARα was ubiquitously expressed in the nuclei of mammary epithelial cells (see Additional file 8). It appears, therefore, that the effects of RA are controlled by the restricted expression of ALDH isoforms in small islands of epithelial or stromal cells.

We demonstrated that ER+ cells are generated by ALDE+ cells. We further investigated whether ALDH KD affected generation of ER+ cells by assessing representation of ER+ cells in mammospheres after silencing ALDH1A1 and ALDH1A3 with shRNAs. We detected significantly fewer ER+ cells in spheres derived from ALDH1A1 KD cells compared with spheres from nontargeting controls or ALDH1A3 KD cells (Figure 4D,E). Given that ER has been found to be a target of RA [38], our findings can be due to a direct effect on ER expression. Branching morphogenesis in 3D Matrigel culture was abolished by ALDH1A1 KD in three different mammoplasty samples (Figure 4F), although mammary epithelial cells proliferated in these conditions and formed spheres or structures with very limited branching. Interestingly, RA treatment of the Matrigel culture completely abolished branching morphogenesis of mammary epithelial cells, without blocking their proliferation. It is not clear what accounts for this effect and if it may be an *in vitro* artifact. Together, the effects of ALDH1A1 KD on mammosphere formation and branching morphogenesis demonstrate that this isoform has a functional role in regulating human mammary stem/progenitor cell proliferation and/or early differentiation.

## Discussion

Numerous studies in animals and humans have demonstrated that development of the mammary gland is dependent on ovarian steroid hormones. It is well established that in mice, these hormones achieve their effects on mammary morphogenesis through paracrine signaling [9,39,40]. The identification and characterization of stem cells from the mouse mammary gland revealed that these cells do not express ER or PR, but respond to stimulation by steroid hormones through paracrine signaling [4-8]. Indirect evidence suggests that this may be the case for the human mammary gland as well. Human mammary stem/progenitor cells identified by the phenotype CD49f+/EpCAM–/Lin– generated ER+ cells *in vivo*, on xenotransplantation under the renal capsule of immunodeficient mice [30]. Work from the Kuperwasser group [31] showed that stem/progenitor cells identified by the CD10+/EpCAM– phenotype could generate ER+ cells *in vivo*, in the humanized, cleared-fat-pad mouse model [31]. ER+ cells could also be generated by CD10–/EpCAM+ cells, which had restricted luminal differentiation potential *in vitro*, but a broader lineage potential *in vivo*.

We previously identified a relatively small mammary epithelial cell population with high ALDH enzymatic activity and functional characteristics of stem/progenitor cells [12]. We now show that this cell population can be detected *in situ* by using immunostaining for ALDH1A1 and ALDH1A3 isoforms. ALDH1A3+ cells are found in larger ducts. ALDH1A1+ cells, although apparently situated in the luminal layer of the mammary ducts, have a particular localization at the branching point of intralobular ductules. This latter subpopulation of cells is relatively small and does not display clear lineage markers of differentiation ([12] and unpublished observations). Given the intralobular localization of ALDH1A1+ cells and the ductal localization of ALDH1A3+ cells, we speculate that ALDH1A3 may be associated with a progenitor cell subpopulation active before adulthood, during duct formation and elongation, and less proliferative in the adult breast. ALDH1A1 may be associated with an intralobular stem/progenitor cell population active in the adult breast. This would be in line with previous reports of sequential spatial and temporal activity of ALDH isoforms in developing tissues [41-43].

By using stem/progenitor cell markers based on ALDH activity and detection of this cell population *in situ*, we provide additional evidence that mammary stem cells are ER–. We also demonstrate in functional assays *in vitro* and *in vivo* that ALDE+/ER– mammary stem/progenitor cells give rise to ER+ progenitor cells, which in turn can generate cells committed to the luminal lineage. It is known that only a small percentage of ER+ cells express markers of proliferation [3,34]. Studies using surrogate markers of ER showed that these cells can function as progenitors of the luminal lineage [26,44]. We now provide direct evidence that a subset of ER+ cells from the normal breast can proliferate and generate progeny with luminal lineage characteristics, a finding with potential implications for the origin of ER+ breast cancers.

Numerous studies concur that, in breast cancer and in cancers from other tissues, ALDH activity and/or expression identifies the subpopulation with highest tumorigenicity and metastatic potential [12,21,45,46]. General agreement also exists that ALDH1A1 correlates with ER– status in breast cancers and is an independent predictor of poor clinical outcome [12,22,27,45].

These observations, together with the findings of our study, indicate that properties of normal stem/progenitor cells may be inherited by cancer stem cells. However, the ability of ALDH to identify stem/progenitor cells in the normal mammary gland has been disputed [24]. Our finding that downregulation of the two isoforms ALDH1A1 and ALDH1A3 affects proliferation of normal human mammary epithelial cells in suspension culture constitutes additional supporting evidence for our initial reports that ALDH activity associates with stem/progenitor cells in normal breast tissue. ALDH1A1 KD had a more marked effect than ALDH1A3 KD on proliferation of mammary epithelial cells in suspension, consistent with our speculation that it represents the active isoform in the adult breast. Because the majority of breast cancers originate in the lobular part of the mammary ductal tree and only a small percentage originate in bigger ducts, this ALDH1A1+ cell population with stem/progenitor characteristics may be significant for cancer initiation.

Consistent with this notion, in BRCA1 healthy carriers, loss of heterozygosity at the BRCA1 locus was found specifically in these epithelial cells expressing ALDH1A1 and was not present in the rest of the epithelium or in surrounding stroma [32].

Analysis of sections through normal mammary epithelium tissue co-stained with ALDH1A1 and ER showed high ER expression in cells adjacent to ALDH1A1+ cells. Taken together with the functional results presented herein, this observation suggests either that ER+ cells are early progenies of ALDH+ cells and/or that their proximity is necessary for conveying paracrine signals. ALDH enzymes convert retinol to RA, a molecule that functions as a diffusible morphogen during embryonic development of a variety of tissues. Spatial and temporal distribution of different ALDH isoforms governs normal differentiation and patterning by establishing cellular fields with gradients of RA [41,47]. RA regulates cell proliferation and differentiation, through binding to nuclear retinoic acid receptors (RAR) α, β, and γ, ubiquitously expressed in the normal breast. Interestingly, ER is a target of RA, which upregulates its expression. Moreover, RARα and ER share a majority of specific DNA-binding sites, as shown in a comprehensive study by Hua *et al*. [38]. RARα is also a target of estrogen [48].

**Figure 5:**
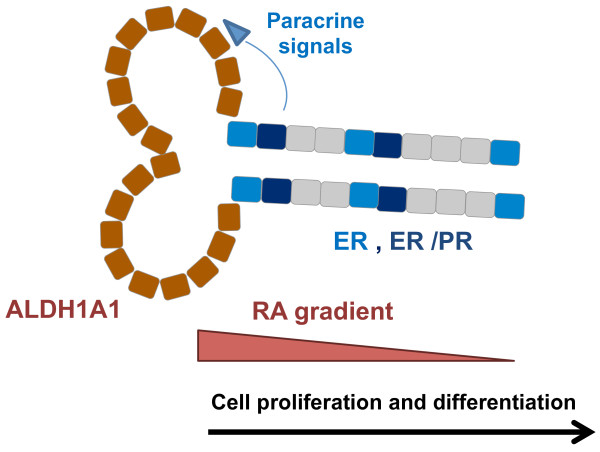
**Proposed model for the contribution of ALDH1A1 and ER+/PR+ cells to the growth of human mammary epithelium.** ALDH1A1+ cells present at branching points and terminal end of intralobular ductules, generate lineage restricted luminal and myoepithelial progenitors. ER+/PR+ cells represent early progenies, with dual role as “sensory” cells that convey paracrine signals to ER– progenitor cells and progenitor cells that generate the luminal lineage. The combined effect of diffusible, short-range morphogens (that is, RA and Wnt) dictates the fate of mammary epithelial cells. These events are coordinated with systemic developmental requirements, being subjected to estrogen and progesterone control.

We propose a model in which ALDH1A1+ stem/progenitor cells generate the cells that compose the layers of intralobular ductules (Figure 5). Early progenitors arising from ALDH1A1+/ER– cells express ER, possibly fulfilling also the role of “niche cells,” as put forward by Brisken and Duss [49]. Upon stimulation with estrogen during the follicular phase of the menstrual cycle, PR is upregulated. Increased levels of progesterone during the luteal phase trigger paracrine signaling that lasts as long as the levels of ovarian hormones are high and ER/PR expression persists, stimulating proliferation of adjacent cells. The ALDH1A1+ cells synthesize RA, which diffuses away from these cells, establishing a gradient controlled by distance and antagonist factors, as described in a variety of other tissues [47]. It is likely that effects of this RA gradient combined with those of other short-range signals initiated by ER+/PR+ cells, such as Wnt and RANKL, dictate cell fate and result in the pleomorphic outcomes attributed to RA: cycle arrest or proliferation and differentiation. The levels of ovarian hormones coordinate these effects with systemic developmental requirements. Thus, sustained high levels of progesterone during pregnancy will maintain a higher rate of cell proliferation in the stem/progenitor compartment of the mammary epithelium, through a positive-feedback loop. Outside pregnancy, only a small expansion of the stem/progenitor cell population will occur during each luteal phase of the menstrual cycle. In nulliparous women, cumulative effects of these events over time may eventually lead to an increase in breast cancer risk.

## Conclusions

This study identifies isoforms ALDH1A1 and ALDH1A3 as being associated with the enzymatically active ALDE+ cell subpopulation of normal human mammary epithelial cells. We present direct evidence that ALDE+ stem/progenitor cells are ER– and that they generate ER+ cell progeny. ER+ cells can function as progenitor cells, giving rise to cells bearing markers of luminal lineage differentiation. ALDH1A1, through its function in RA metabolism, plays a role in proliferation and differentiation of mammary progenitor cells.

## Abbreviations

ALDE: ALDEFLUOR; ALDH: aldehyde dehydrogenase; ATRA: all-*trans* retinoic acid; CK: cytokeratin; ER: estrogen receptor; IHC: immunohistochemistry; KD: knockdown; PI: propidium iodide; PR: progesterone receptor; RA: retinoic acid; RAR: retinoic acid receptor; SMA: smooth muscle actin; WB: Western blot.

## Competing interests

The authors declare that they have no competing interests.

## Authors’ contributions

GH performed experiments, analyzed data, and drafted the manuscript. SL performed experiments and analyzed data. CG, MHH, RM, BB, IS, PG, and SB performed experiments. VR supported tissue procurement and patient data collection. ADP supported conception and design, tissue procurement, and data collection. SEP supported evaluation of immunostainings. GD designed the study, performed experiments, analyzed data, and drafted the manuscript. All authors read and approved the final manuscript.

## Supplementary Material

Additional file 1 **Figure showing specificity for ALDH1A1 and ALDH1A3 antibodies used in immunostainings.** Breast cancer cell lines Cal51 and SUM149 that are positive for ALDH isoforms ALDH1A1 and ALDH1A3, respectively, were transfected with shRNAs for these two isoforms and nontargeting control. Immunostainings with antibodies against ALDH1A1 and ALDH1A3, respectively, were performed 48 hours after transfection. Scale bar = 50 μm. Click here for file

Additional file 2 **Figure showing quantification of ALDH1A1+ and ALDH1A3+ cells in normal breast epithelium. (A)** Tissue sections from five different mammoplasty samples were immunostained for ALDH1A1 and/or ALDH1A3. Example of one section where areas of epithelium are marked. **(B)** ALDH1A1+ cells (red staining, indicated with red arrow), ALDH1A3+ cells (DAB, brown arrow) and total number of cells in each nonoverlapping area were counted. **(C)** Table with quantitative data for cells positive for ALDEFLUOR (ALDE), as determined by flow cytometry, as well as ALDH1A1 and ALDH1A3, as determined by immunostaining in five different mammoplasty samples. Click here for file

Additional file 3 **Figure showing immersed analysis of ER expression in ALDH+ human mammary epithelial cells. (A)** ALDE+ and ALDE– primary human mammary epithelial cells separated by with ACS were immunostained for ER (FITC) and reanalyzed with flow cytometry. ALDE– cells contained 27.8% ER+ cells (left panel), whereas ALDE+ cells did not express ER above background level (right panel, 3.8% of ALDE+ population, 0.002% of the total population). **(B)** Breast cancer cell lines SUM44 (ER+ cell line) and SUM149 (ER– cell line) were used as positive (left panel) and negative (right panel) control for ER expression. The 3.8% positive cells detected with flow cytometry in the ALDE+ cell population (A) represent background staining, as indicated by the presence of 5.1% ER+ cells in SUM149 ER– breast cancer cells, which was similarly immunostained and similarly gated for flow-cytometry analysis. **(C)** Immunostaining for ER on ALDE-sorted cells showed ER+ cells in the ALDE– population, but not in the ALDE+ cell population. **(D,E)** Double staining for ALDH1A3 and ER on normal breast sections show no colocalization. **(F-I)** Double staining for ALDH1A1 and ER on normal breast sections showing representative areas with ERlow/ALDH1A1+ cells (arrows) in two different mammoplasty samples **(H, I)**. ERhigh/ALDH1A1– cells in the same sections are indicated with arrowheads. **(J)** Quantitative assessment of ERlow/ALDH1A1+ cells in normal breast samples revealed a small percentage of double-positive cells only in three of 11 samples. Scale bar = 50 μm. Click here for file

Additional file 4 **Figure showing strategy for identification and isolation of ER+ and ER– cells from normal mammary epithelium. (A, B)** Diagram of experimental steps and reporter construct used for the separation of ER+ and ER– cells. **(C)** Level of ER expression as reported by level of GFP expression in MCF7 ER+ breast cancer cells, MDA-MB-231 ER– breast cancer cells and primary normal mammary epithelial cells (HMEC). **(D)** Immunostaining for ER expression on cytospins from GFP-sorted cells transduced with the Ade 25 ERE Pr GFP construct. Representative images of ER– cells (upper panel) and ER+ cells (lower panel) after separation with the reporter system. Nuclei were detected with PI staining. GFP+ cells contained 95% ER+ cells by immunostaining, and GFP– cells contained 2% ER+ cells. Click here for file

Additional file 5 **Figure showing double staining of mammospheres for ER and Ki67.** Mammosphere sections were double stained for ER (green) and proliferation marker Ki67 (red). White arrows indicate double-positive cells. Scale bar = 25 μm. Click here for file

Additional file 6 Table showing outgrowth potential of normal mammary epithelial cell subpopulations sorted for ER in the humanized fat pad of NOD/scid mice. Click here for file

Additional file 7 **Figure showing primary and secondary mammosphere formation after shRNA knockdown of ALDH1A1. (A)** Primary sphere formation after ALDH1A1 KD with two different shRNA constructs (9 and 10) as well as using a pool of these two shRNAs (9+10). **(B)** Primary and secondary sphere formation after ALDH1A1 KD with combined shRNAs #9 and #10. *P* values given are compared with NT control and were calculated by using a two-tailed *t* test. Click here for file

Additional file 8 **Figure showing RARα staining in normal breast.** Nuclear RARα was expressed in the vast majority of breast epithelial and stromal cells, although occasional negative nuclei were detected in both epithelium and stroma (arrows). Scale bar = 100 μm. Click here for file
