# Supplementary figures and images for: Aldehyde dehydrogenase and estrogen receptor define a hierarchy of cellular differentiation in the normal human mammary epithelium
Source: Breast Cancer Res. 2014 May 27;16(3):R52. doi: 10.1186/bcr3663 (PMC4095680; doi:10.1186/bcr3663)

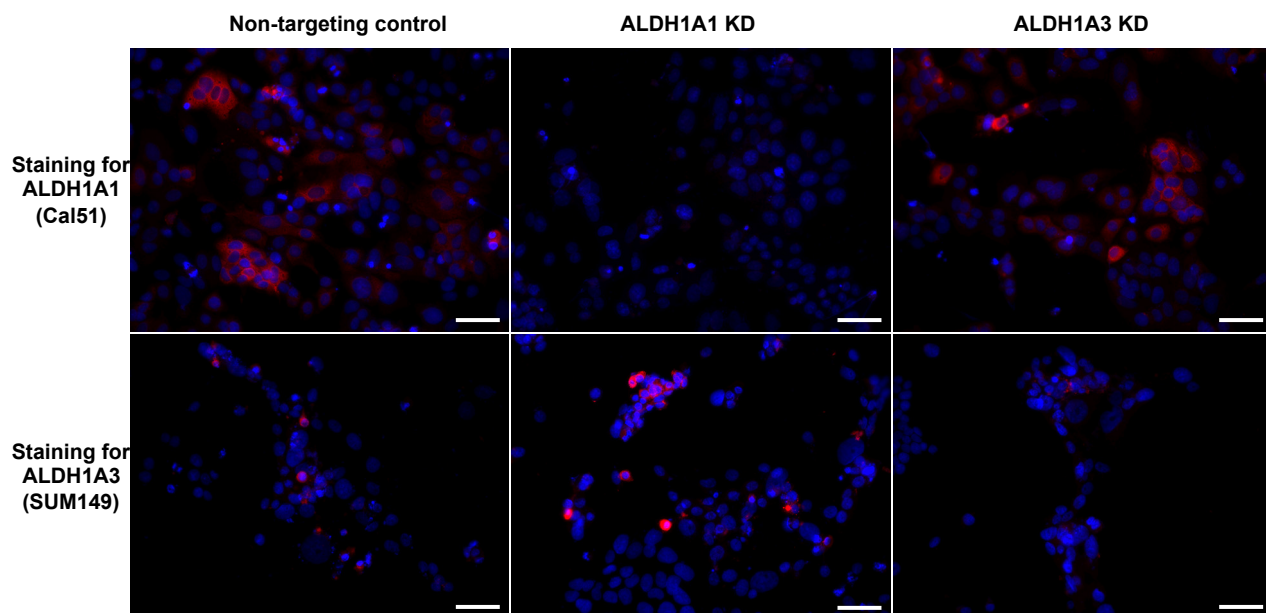

Supplement: Additional file 1 — Figure showing specificity for ALDH1A1 and ALDH1A3 antibodies used in immunostainings. Breast cancer cell lines Cal51 and SUM149 that are positive for ALDH isoforms ALDH1A1 and ALDH1A3, respectively, were transfected with shRNAs for these two isoforms and nontargeting control. Immunostainings with antibodies against ALDH1A1 and ALDH1A3, respectively, were performed 48 hours after transfection. Scale bar = 50 μm. [file bcr3663-S1.pdf]

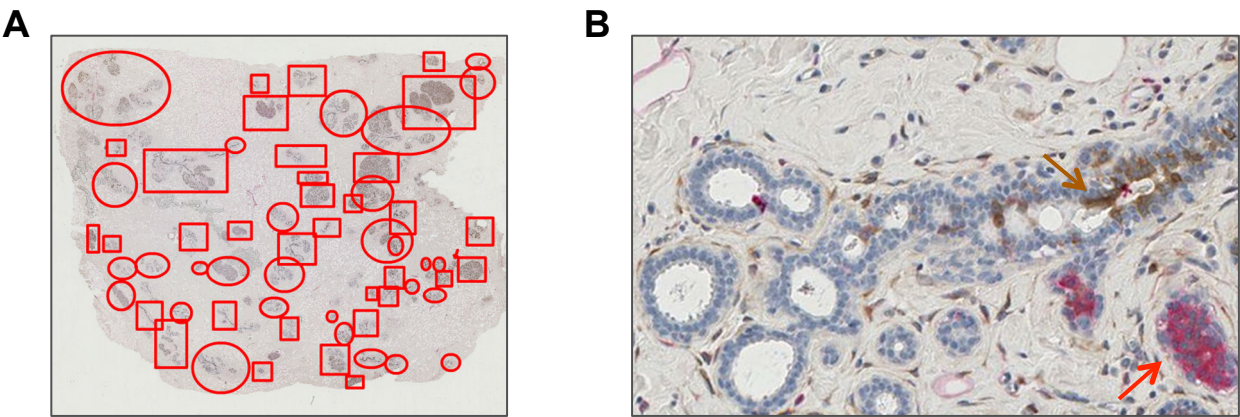

**C**

| Sample  | ALDE (%) | ALDH1A1 (%) | ALDH1A3 (%) |
|---------|----------|-------------|-------------|
| BN004   | 6.7      | 4.8         | 1.3         |
| BN010   | 2.3      | 1.0         | N/A         |
| BN017   | 6.9      | 0.4         | 6.0         |
| BN024   | 1.7      | <0.1        | <0.1        |
| BN036   | N/A      | 1.5         | 0.8         |
| Average | 4.4      | 1.4         | 2.3         |

Supplement: Additional file 2 — Figure showing quantification of ALDH1A1+ and ALDH1A3+ cells in normal breast epithelium. (A) Tissue sections from five different mammoplasty samples were immunostained for ALDH1A1 and/or ALDH1A3. Example of one section where areas of epithelium are marked. (B) ALDH1A1+ cells (red staining, indicated with red arrow), ALDH1A3+ cells (DAB, brown arrow) and total number of cells in each nonoverlapping area were counted. (C) Table with quantitative data for cells positive for ALDEFLUOR (ALDE), as determined by flow cytometry, as well as ALDH1A1 and ALDH1A3, as determined by immunostaining in five different mammoplasty samples. [file bcr3663-S2.pdf]

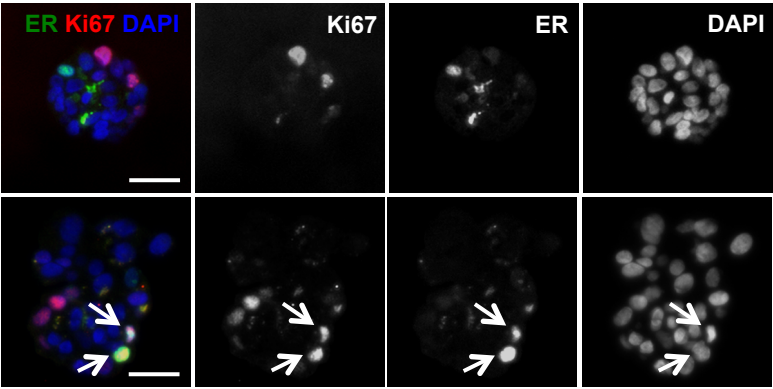

Supplement: Additional file 5 — Figure showing double staining of mammospheres for ER and Ki67. Mammosphere sections were double stained for ER (green) and proliferation marker Ki67 (red). White arrows indicate double-positive cells. Scale bar = 25 μm. [file bcr3663-S5.pdf]

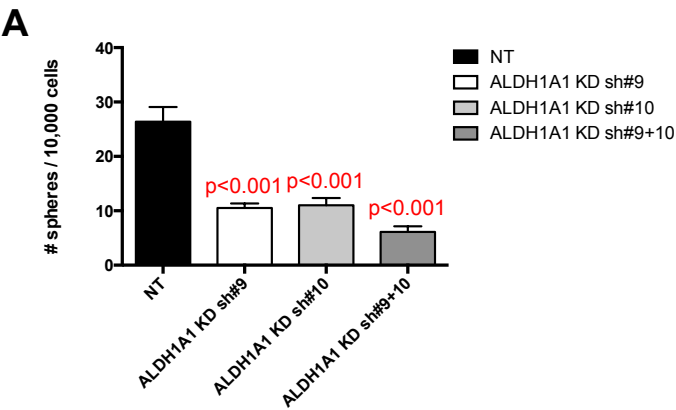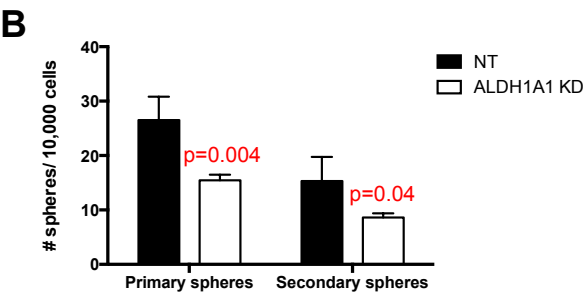

Supplement: Additional file 7 — Figure showing primary and secondary mammosphere formation after shRNA knockdown of ALDH1A1. (A) Primary sphere formation after ALDH1A1 KD with two different shRNA constructs (9 and 10) as well as using a pool of these two shRNAs (9+10). (B) Primary and secondary sphere formation after ALDH1A1 KD with combined shRNAs #9 and #10. P values given are compared with NT control and were calculated by using a two-tailed t test. [file bcr3663-S7.pdf]

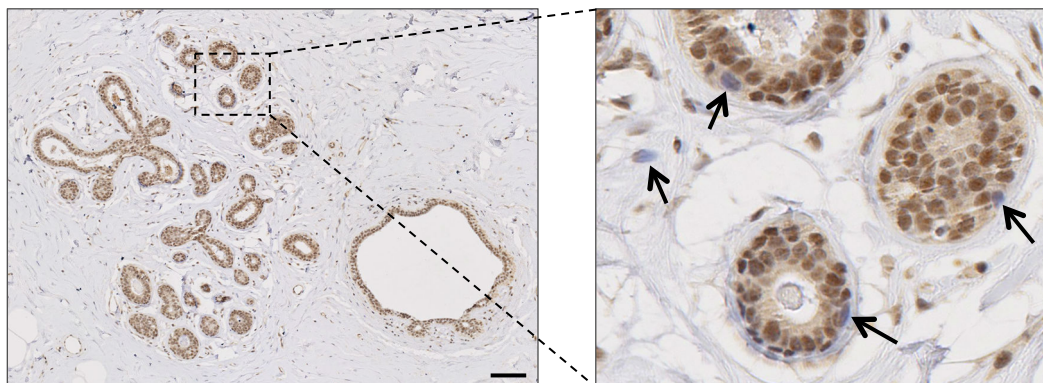

Supplement: Additional file 8 — Figure showing RARα staining in normal breast. Nuclear RARα was expressed in the vast majority of breast epithelial and stromal cells, although occasional negative nuclei were detected in both epithelium and stroma (arrows). Scale bar = 100 μm. [file bcr3663-S8.pdf]
