# Supplementary material for: Aldehyde dehydrogenase and estrogen receptor define a hierarchy of cellular differentiation in the normal human mammary epithelium
Source: Breast Cancer Res. 2014 May 27;16(3):R52. doi: 10.1186/bcr3663 (PMC4095680; doi:10.1186/bcr3663)
Supplement: Additional file 3 — Figure showing immersed analysis of ER expression in ALDH+ human mammary epithelial cells. (A) ALDE+ and ALDE– primary human mammary epithelial cells separated by with ACS were immunostained for ER (FITC) and reanalyzed with flow cytometry. ALDE– cells contained 27.8% ER+ cells (left panel), whereas ALDE+ cells did not express ER above background level (right panel, 3.8% of ALDE+ population, 0.002% of the total population). (B) Breast cancer cell lines SUM44 (ER+ cell line) and SUM149 (ER– cell line) were used as positive (left panel) and negative (right panel) control for ER expression. The 3.8% positive cells detected with flow cytometry in the ALDE+ cell population (A) represent background staining, as indicated by the presence of 5.1% ER+ cells in SUM149 ER– breast cancer cells, which was similarly immunostained and similarly gated for flow-cytometry analysis. (C) Immunostaining for ER on ALDE-sorted cells showed ER+ cells in the ALDE– population, but not in the ALDE+ cell population. (D,E) Double staining for ALDH1A3 and ER on normal breast sections show no colocalization. (F-I) Double staining for ALDH1A1 and ER on normal breast sections showing representative areas with ERlow/ALDH1A1+ cells (arrows) in two different mammoplasty samples (H, I). ERhigh/ALDH1A1– cells in the same sections are indicated with arrowheads. (J) Quantitative assessment of ERlow/ALDH1A1+ cells in normal breast samples revealed a small percentage of double-positive cells only in three of 11 samples. Scale bar = 50 μm. [file bcr3663-S3.pdf]

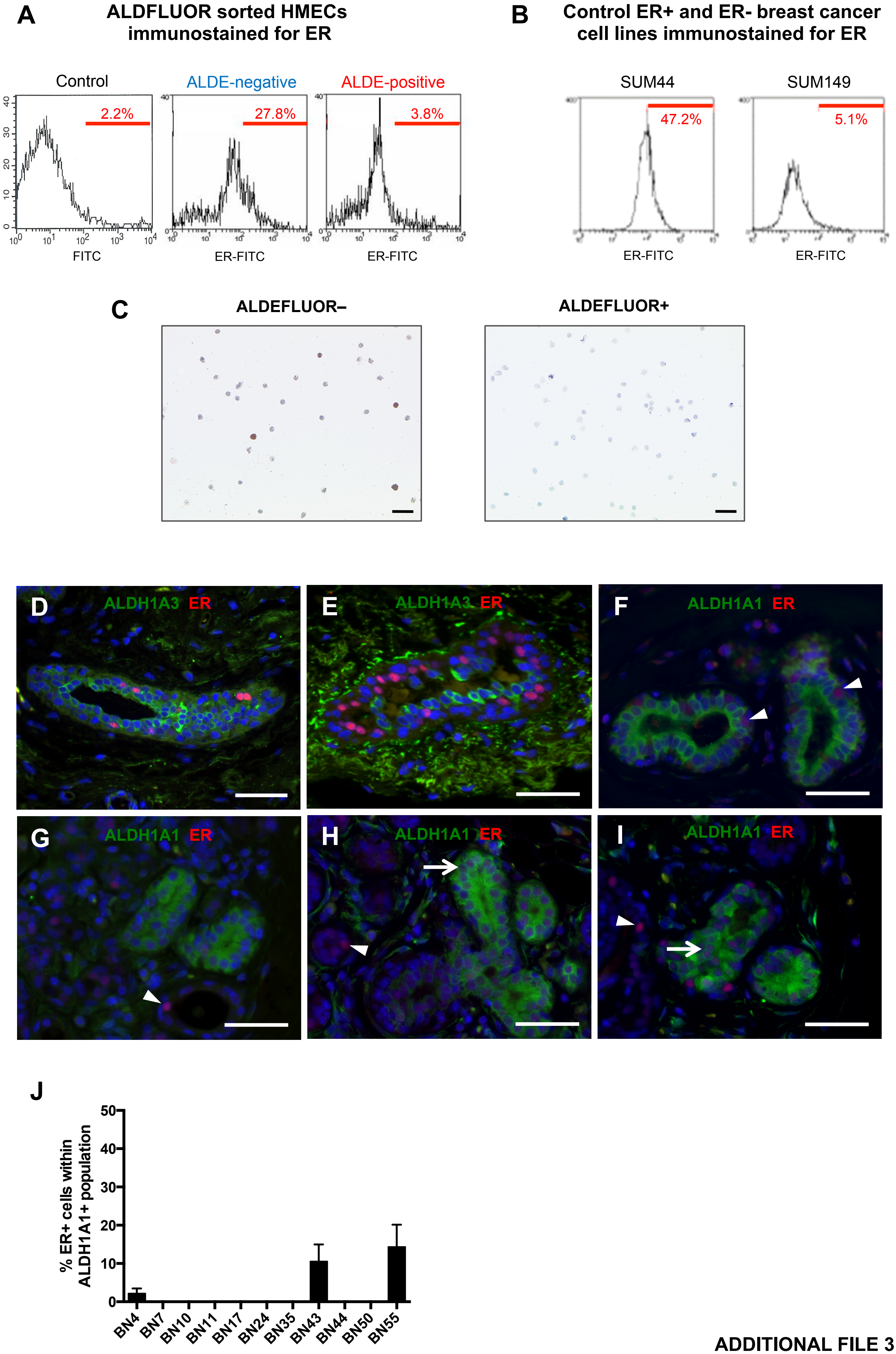

ADDITIONAL FILE 3
