# Supplementary material for: Aldehyde dehydrogenase and estrogen receptor define a hierarchy of cellular differentiation in the normal human mammary epithelium
Source: Breast Cancer Res. 2014 May 27;16(3):R52. doi: 10.1186/bcr3663 (PMC4095680; doi:10.1186/bcr3663)
Supplement: Additional file 4 — Figure showing strategy for identification and isolation of ER+ and ER– cells from normal mammary epithelium. (A, B) Diagram of experimental steps and reporter construct used for the separation of ER+ and ER– cells. (C) Level of ER expression as reported by level of GFP expression in MCF7 ER+ breast cancer cells, MDA-MB-231 ER– breast cancer cells and primary normal mammary epithelial cells (HMEC). (D) Immunostaining for ER expression on cytospins from GFP-sorted cells transduced with the Ade 25 ERE Pr GFP construct. Representative images of ER– cells (upper panel) and ER+ cells (lower panel) after separation with the reporter system. Nuclei were detected with PI staining. GFP+ cells contained 95% ER+ cells by immunostaining, and GFP– cells contained 2% ER+ cells. [file bcr3663-S4.pdf]

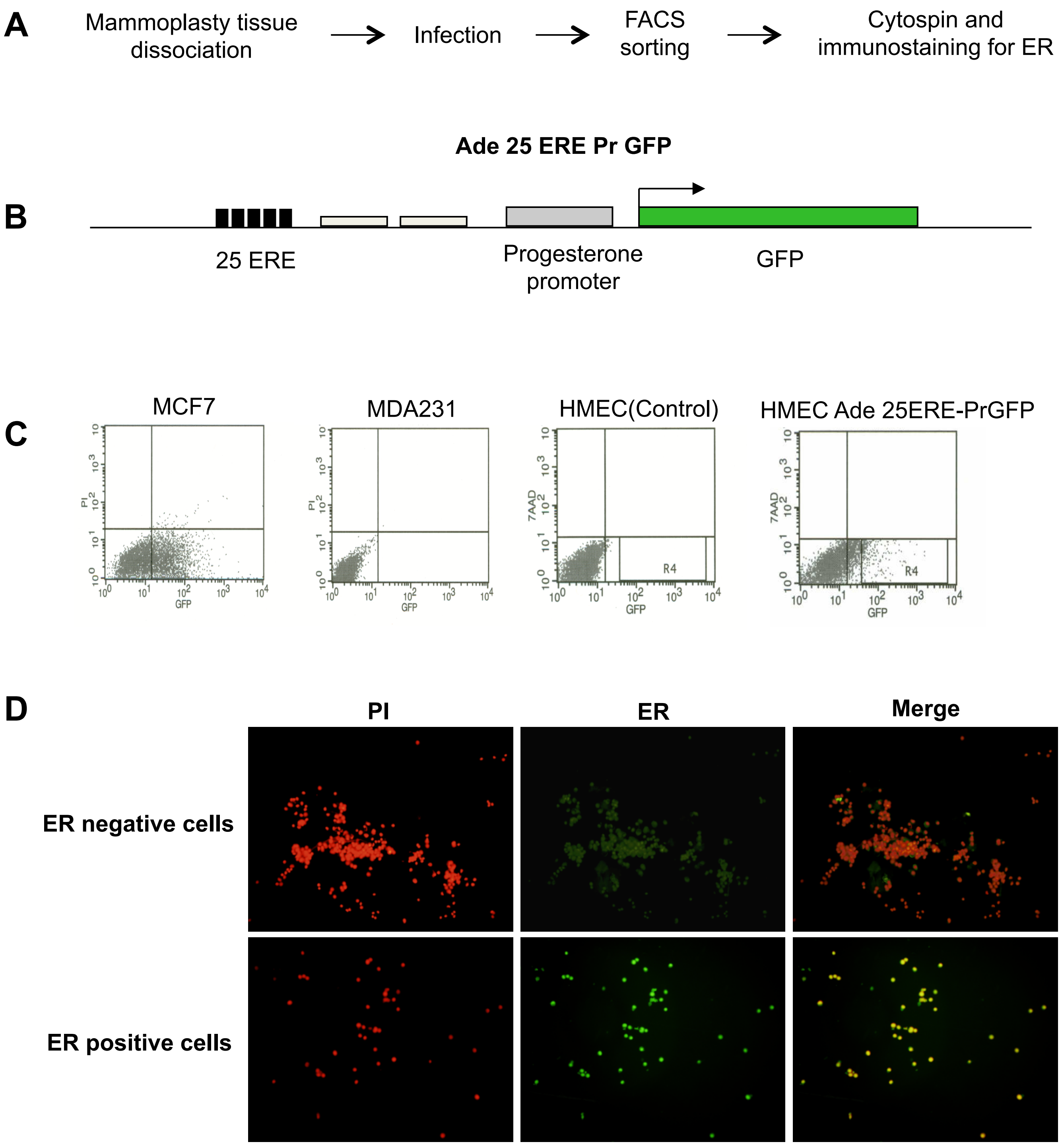

ADDITIONAL FILE 4
