# Supplementary material for: Aldehyde dehydrogenase and estrogen receptor define a hierarchy of cellular differentiation in the normal human mammary epithelium
Source: Breast Cancer Res. 2014 May 27;16(3):R52. doi: 10.1186/bcr3663 (PMC4095680; doi:10.1186/bcr3663)
Supplement: Additional file 6 — Table showing outgrowth potential of normal mammary epithelial cell subpopulations sorted for ER in the humanized fat pad of NOD/scid mice. [file bcr3663-S6.pdf]

Outgrowth potential of normal mammary epithelial cell subpopulations sorted for ER in the humanized fat pad of NOD/scid mice.

	Outgrowth/injections			
	$8x10^5$	$2.5x10^5$	$1.5x10^5$	$2.5x10^4$
**ER-positive**	1/1	2/2	4/4	1/4
**ER-negative**	1/1	2/2	4/4	4/4
**Unsorted**	1/1	2/2	4/4	4/4
